# A Practical Demonstration

**Published:** 1920-02-21

**Authors:** 


### A Practical Demonstration.

### The Public Pharmacists' Association.

The Annual General Meeting was held at St. Bride Institute, London, on January 28, Mr. A. H. Jenkin in the chair. Four new members were elected—viz., Miss C. Marshall, Messrs. J. G. Fry (West Ham), R. Fouracre (Westminster Hospital), and R. V. Gurd (Colney Hatch Mental Hospital). There was a good attendance of members to hear the annual report of the Council for 1919 read. This showed that much time and energy had been spent by the Executive on matters concerning members who act as pharmacists to voluntary hospitals and public institutions. The full report will be circulated in due course.

The officers elected for 1920 were: President, T. H. W. Idris, Esq., J.P.; Chairman, J. L. P. Hollingworth, F.L.S. (Children's Hospital, Chelsea); Vice-Chairman, A. Howell; Hon. Secretary and Treasurer, G. W. Gibson (St. Pancras); Hon. Secretary "Poor-Law Section," R. W. Lindsey; Assistant Secretary, A. J. Gibbons (St. Thomas's); Council, Misses Andrews, Gilliatt, Messrs. Cairns (L.C.C.), France (Westminster), Jenkin, Moore (St. Pancras), Bullen (H.M. Prison), Skinner (Great Northern).

Practically all the public services are thus represented. At the conclusion of the formal business Mr. Jenkin gave a demonstration on "Dispensing Time Savers." He showed many mechanical devices for expediting the work of a dispensary both for the laboratory and out-patient departments, an improvised label damper constructed of a glass stopper, lint, and an ointment jar showed what ingenuity could accomplish, a clip for labels, rapid filter apparatus, and many other items of practical interest. In view of the fact that rebate on alcohol used in medicinal preparations is now granted at the rate of £2 18s. per gallon, the Secretary gave some details as to the method of making up the records to the satisfaction of the Excise Authorities, and, although it appears a formidable business, the explanatory details proved that no elaborate calculations are required. This rebate, when claimed, should prove a help in keeping down the drug bill. The next meeting of the Associations is to be held at St. Bride Institute, London, E.C., on Wednesday, February 25.

